# Lower Urinary Tract Diseases in Guinea Pigs: A 14-Year Retrospective Study (2004–2018)

**DOI:** 10.3390/ani13010112

**Published:** 2022-12-28

**Authors:** Salomé Azevedo, Bairbre O’Malley, Claire Greene, Helena Moran, Tomás Rodrigues Magalhães, Felisbina Luísa Queiroga

**Affiliations:** 1 Department of Veterinary Sciences, University of Trás-os-Montes and Alto Douro, Quinta dos Prados, 5000-801 Vila Real, Portugal; 2 Bairbre O’Malley Veterinary Hospital, 7 Kilmantain Place, A98 NY03 Bray, Ireland; 3 Animal and Veterinary Research Centre (CECAV), University of Trás-os-Montes and Alto Douro, Quinta dos Prados, 5000-801 Vila Real, Portugal; 4 Associate Laboratory for Animal and Veterinary Sciences (AL4AnimalS), University of Trás-os-Montes and Alto Douro, Quinta dos Prados, 5000-801 Vila Real, Portugal; 5 Center for the Study of Animal Sciences, CECA-ICETA, University of Porto, 4200-465 Porto, Portugal

**Keywords:** guinea pigs, urolithiasis, cystitis, urinary tract infection, urinary disease

## Abstract

**Simple Summary:**

The clinical records of all guinea pigs diagnosed with a lower urinary tract disease in a single veterinary hospital, over a period of 14 years (2004–2018), were searched in order to characterize this population and investigate the potential association between the different features relating to the animal and the clinical approach toward them. A total of 117 clinical cases were identified, corresponding to 57 animals. The formation of stones in the urinary tract (urolithiasis) was the most common diagnosis (*n* = 52; 44.4%), followed by bladder inflammation (cystitis) and/or a urinary tract infection (UTI). Several associations were identified, showing that female guinea pigs were more likely than the male ones to have a previous family history of urinary disease, to present abnormal micturition signs at admission, and to have recurrence. Moreover, males were more prone to urolithiasis and females to cystitis/UTI, and animals diagnosed with cystitis/UTI frequently had more clinical urinary signs and abdominal pain on palpation compared to those diagnosed with urolithiasis. Finally, the use of potassium citrate and the urethrotomy approach were associated with a better therapeutic response. Further studies are needed in larger populations of guinea pigs to confirm the present findings, especially as some of them were described for the first time.

**Abstract:**

The clinical records of all guinea pigs diagnosed with a lower urinary tract disease in a single veterinary hospital, over a period of 14 years (2004–2018), were retrospectively searched in order to characterize this population and investigate the potential association between the epidemiological and clinical variables. A total of 117 clinical cases were identified, corresponding to 57 animals. Urolithiasis was the most common diagnosis (*n* = 52; 44.4%), followed by cystitis and/or a urinary tract infection (UTI). Several statistically significant associations (*p* < 0.05) were found between different variables, showing that female guinea pigs were more likely than the male ones to have a previous family history of urinary disease, to present dysuria and stranguria at admission, and to suffer recurrence. Moreover, males were more prone to urolithiasis and females to cystitis/UTI, and animals diagnosed with cystitis/UTI frequently had more clinical urinary signs and abdominal pain on palpation compared to those diagnosed with urolithiasis. Finally, the use of potassium citrate and the urethrotomy approach were associated with a better therapeutic response. Further studies are needed in larger populations of guinea pigs to confirm the present findings, especially as some of them were described for the first time.

## 1. Introduction

The guinea pig (*Cavia porcellus*) belongs to the *Caviidae* family, which in turn is part of the Order *Rodentia* and Suborder *Hystricomorpha* [1]. Despite originally being considered a laboratory animal, it has increasingly been chosen as a pet, which promotes its greater coexistence with humans and an increase in the demand for appropriate veterinary care [2,3].

Nowadays, more diseases have been diagnosed in this species, as owners of these animals seem to be more aware of and alert to detecting changes in their health status. For example, among the bacterial infections that affect guinea pigs, urinary tract infections (UTI) are the most frequently reported ones [4]. However, UTIs are just one of the urinary disorders that affect this species, as urolithiasis and cystitis are also common in middle-aged to older cavies [5,6].

A variety of urinary clinical signs are associated with these disorders, such as dysuria, stranguria, hematuria, anuria, pollakiuria, and vocalization when attempting to urinate. However, other nonspecific and vague signs have also been described, such as lethargy, teeth grinding, anorexia, weight loss, and hunched posture [2,6,7,8]. Therefore, diagnosis should not only be based on the clinical presentation, but should always consider other factors as well, such as the medical history, the physical examination findings, and the results of complementary tests, particularly radiography and ultrasonography [2,7].

The treatment depends on the etiology of the urinary disorder, but it generally includes the use of analgesics to control pain, antibiotic therapy in case of a positive bacterial culture, and diet modification to reduce the levels of oxalate and calcium that create a predisposition to the formation of uroliths [5]. In animals with urolithiasis, surgery is commonly required, given the inefficacy of medical treatment to dissolve the uroliths [8,9].

The aim of this retrospective study was to characterize a population of guinea pigs diagnosed with a lower urinary tract disease regarding their clinical presentation, as well as the diagnostic and therapeutic approach to them, and to investigate a potential association between the clinical variables and sex, diagnosis, and response to treatment.

## 2. Materials and Methods

### 2.1. Case Selection and Data Acquisition

The clinical records of all guinea pigs diagnosed with lower urinary tract disease at Bairbre O’Malley Veterinary Hospital were searched, and all cases of urolithiasis, cystitis, and urinary tract infection (UTI) were retrieved for further analysis. The time frame defined was from 1 June 2004 to 31 June 2018, allowing for the study of a 14-year period in total.

It should be noted that, if the animal had more than one episode of lower urinary tract disease, each clinical episode was recorded independently, as long as it was a different diagnosis and it was not related to the previous one (e.g., a recurrence or subsequent complication).

Regarding the data acquired, information was obtained concerning several variables of interest to the study, such as age, sex, reproductive status, type of medical consultation, disease recurrence, diet, reason for presentation, clinical signs (dysuria, hematuria, and stranguria), physical examination findings (abdominal pain and presence of urolith on palpation), diagnostic tests (urinalysis type 1 and type 2, bacteriological culture, radiographs, ultrasound, and blood testing), presence of uroliths and their location, diagnosis, and therapeutic features (type of treatment, type of surgery, use of glucosamine and potassium citrate, and treatment response). 

### 2.2. Patient, Diagnostic and Therapeutic Features

For some variables, the data collected were grouped into different categories to allow for a more objective assessment. For example, in relation to the diet, two categories were considered based on the type of hay and pellets the cavies ate. A low-calcium diet was considered when cavies ate oat, timothy, or grass hay and pellets, and a normal/high calcium diet was considered when cavies did not eat this type of food.

Regarding the reason for presentation, four categories were defined: “urinary signs” (dysuria, hematuria, stranguria, abdominal pain, or presence of urolith on palpation), “nonspecific clinical signs” (weight loss, gut stasis, anorexia, diarrhea, lethargy, polyuria, and polydipsia), “urinary signs and nonspecific clinical signs,” and “shock.” The last category was reserved for animals that presented with abnormal mental status and were in need of emergency treatment.

The diagnosis of each clinical episode was classified into one of two categories: “urolithiasis” (when a urolith was detected) or “cystitis/UTI” (when urolithiasis was ruled out by radiography and abdominal ultrasound and an inflammatory and/or infectious process was detected in the urinary bladder).

The location of uroliths was defined in four anatomical regions of the urinary tract: “ureter,” “bladder,” “urethra,” and “ureter and urethra,” with the latter being used in cases where there was more than one urolith and whenever it affected regions at the same time. The type of surgery performed to remove them was also defined as “cystotomy,” “urethrotomy,” or “urethrotomy + cystotomy.”

### 2.3. Statistical Analysis

The SPSS^®^ (Statistical Package for the Social Sciences) software (Version 24.0; IBM Corp©) was used for statistical analysis. Categorial variables were expressed in absolute and relative frequencies and their potential associations were investigated by the Chi-square test and the Fisher’s exact test, as appropriated. A univariate analysis was conducted, and statistical significance was defined when *p* < 0.05.

## 3. Results

One hundred and seventeen clinical episodes were identified, corresponding to 57 guinea pigs in total. All these cases met the previously defined eligibility criteria and were therefore included for further analysis.

### 3.1. Patient Signalment

Considering the population of 117 cases, 68 were females (58.1%) and 49 were males (41.9%), and the median age at presentation was 3 years old, ranging from 1 to 7 years.

Most of the guinea pigs were intact (*n* = 103; 88.0%) and only 14 (12.0%) were neutered. Among females, 67 were intact (98.5%) and 1 was neutered (1.5%), while in males, 36 were intact (73.5%) and 13 were neutered (26.5%). 

As shown in Table 1, sex showed a statistically significant association with several other features: reproductive condition (*p* < 0.001), disease recurrence (*p* = 0.038), family history of urinary disease (*p* < 0.001), clinical manifestation of dysuria and stranguria (*p* = 0.038 and *p* = 0.038, respectively), and diagnosis (*p* < 0.001). Therefore, there was a greater tendency for females to be intact at diagnosis, to have a recurrence of urinary disease and a family history, and to present with dysuria and stranguria at the time of admission. Moreover, males were more prone to urolithiasis and females to cystitis/UTI.

### 3.2. Diet Habits and Family History of Urinary Disease

Information about the guinea pigs’ diet was available for 82 animals: 46 (56.1%) were fed a diet low in calcium and 36 (43.9%) were fed a diet with normal/high calcium levels. All the guinea pigs were fed a pellet diet, except for one, which was fed a muesli diet. In addition, all the animals had hay and fresh water available and were fed with fresh fruit and vegetables. Unfortunately, the information about their drinking system was not available, preventing the study of its impact on the occurrence of the disease in this population.

When asked for the guinea pig’s family history, in 60 cases (51.3%), it was reported that they had relatives with lower urinary tract disease, and in 57 cases (48.7%), there was no family history identified.

### 3.3. Type and Reason for Consultation and Clinical Presentation

According to the type of consultation, 66 (56.4%) were seen as a first opinion, whereas the remaining 51 (43.6%) were seen as referral cases. Moreover, 62 cases (53.0%) were presented at the hospital due to disease recurrence and 55 (47.0%) were seen following a first episode of the disease. 

Eighty-two animals (70.1%) were admitted only with urinary signs, 18 (15.4%) had urinary signs associated to other nonspecific signs, 16 (13.7%) had nonspecific signs and did not have any clinical manifestations that could indicate a disease relating to the urinary tract, and one cavie arrived already in shock (0.9%). 

Regarding urinary signs, 84 animals showed dysuria and stranguria, 78 presented hematuria, and 72 had abdominal pain upon palpation. In addition, the nonspecific clinical signs included anorexia and weight loss (*n* = 19), polydipsia and polyuria (*n* = 9), diarrhea (*n* = 4), lethargy (*n* = 4), and gut stasis (*n* = 2). 

### 3.4. Complementary Tests

In addition to the clinical presentation, previous medical history, and physical examination, the diagnosis was established based on several complementary exams: a urinalysis (type 1 and 2), a bacteriological culture, a radiographic study, an abdominal ultrasound, a complete blood count, and the serum biochemistry. However, due to the owners’ financial constraints, a full diagnostic work-up was not always performed, and, for some animals, the diagnosis was presumptive. It is also important to mention that the owners frequently rejected a urolith analysis in order to define its composition.

Radiography was the most commonly required diagnostic exam (*n* = 105; 89.7%), followed by urinalysis type I (*n* = 64; 54.7%), urinalysis type II (*n* = 55; 47.0%), blood testing (complete blood count and serum biochemistry; *n* = 12; 10.3%), urine culture (*n* = 9; 7.7%) and abdominal ultrasound (*n* = 6; 5.1%). It should be noted that type 1 urinalysis included the physical and chemical evaluation of the urine through a urine test strip only, while type II urinalysis also included the determination of specific urine gravity and a microscopic examination of the urine sediment.

**Figure 1:**
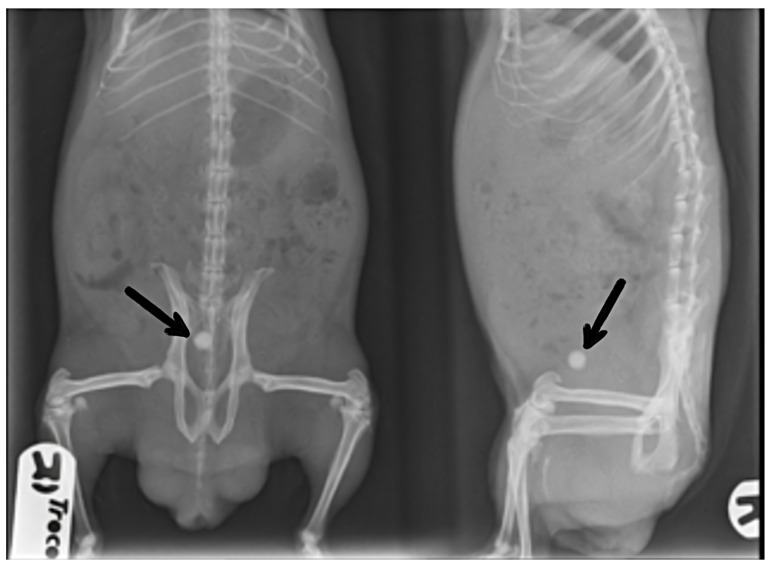
Radiographs of a sedated male guinea pig with an urolith in the bladder (marked with arrows) in ventrodorsal and right latero-lateral projections.

The radiographic study permitted the observation of uroliths in the guinea pig population studied: in 28 cases, the urolith was located in the bladder (Figure 1), with 14 cases of it in the urethra and 7 cases of it in the ureter. In addition, three animals had more than one urolith (one in the urethra and another one in the ureter). In male guinea pigs, most uroliths were located in the bladder (*n* = 23), while in females, most were associated with the urethra (*n* = 13), revealing a significant association between sex and anatomical location (*p* = 0.001; data not presented).

Through urinalysis (type 1 and type 2), several findings were found, such as the presence of protein (*n* = 51), the presence of blood (*n* = 45) and red blood cells (*n* = 29), the presence of white blood cells (*n* = 18), pyuria (*n* = 16), and crystalluria (*n* = 14). The pH value recorded for most cases (*n* = 51) was 9, while in 12 cases it was 8, and in a single case it was 7. Regarding the type of crystals detected in the urine, in seven cases, calcium carbonate and calcium oxalate were both identified, in five cases, calcium carbonate and struvite were simultaneously found, and in the remaining two cases, only one type of crystal was observed (solely calcium carbonate or oxalate crystals). The urine-specific gravity was also measured for these animals, ranging from 1.010 to 1.020. In five out of nine cases, the bacteriological culture was positive, allowing for the identification of the following bacteria: *Pseudomonas* spp. (*n* = 8), *Staphylococcus* spp. (*n* = 5), and *Escherichia coli* (*n* = 3), which shows that more than one bacterial species was isolated in most of the samples collected.

The blood collected from 12 cavies (10.3%) was also processed and the following parameters were measured: packed cell volume (PCV), serum glucose, creatinine, urea, urea/creatinine ratio, total protein, albumin, globulins, albumin to globulin ratio, alanine aminotransferase (ALT), and alkaline phosphatase (ALP). Ten animals showed an increase in serum glucose, globulins, and ALT, while the two others had all normal parameters. 

Finally, a complete abdominal ultrasound was performed in only six animals (5.1%), which permitted the diagnosis of bladder calculi (*n* = 3) and hydronephrosis secondary to ureterolithiasis (*n* = 2), as well as the identification of a thickened bladder wall (*n* = 1).

### 3.5. Diagnosis

Fifty-two cases (44.4%) were diagnosed with urolithiasis: in 50 cases, the definitive diagnosis was achieved through the visualization of the urolith on radiographs, in 1 guinea pig, the urolith was eliminated in the urine during the consultation, and in another case, the diagnosis was reached post-mortem through the direct visualization of several uroliths during the necropsy. The determination of the urolith composition was performed in only 14 of these cases (26.9%). The remaining cases (*n* = 65; 55.6%) were diagnosed as cystitis and/or UTIs.

In addition to a significant relationship with sex, as mentioned above, the diagnosis showed a statistically significant association with the reason for consultation (*p* = 0.002), the clinical manifestation of dysuria, hematuria, and stranguria (*p* = 0.027, *p* < 0.001 and *p* = 0.038, respectively), and abdominal pain (*p* = 0.004) on palpation during the physical examination (Table 2). Thus, there was a greater tendency for animals diagnosed with cystitis and/or UTIs to present these clinical urinary signs at admission, as well as to manifest abdominal pain during the physical examination, compared to those diagnosed with urolithiasis.

### 3.6. Treatment

Eighty-four cases (71.8%) were treated medically, while 33 (28.2%) required surgery. For the animals that required hospitalization (*n* = 93; 79.5%), it was necessary to administer fluids, to control pain with non-steroidal anti-inflammatory drugs (meloxicam or carprofen) and/or analgesics (buprenorphine), and to perform antibiotic therapy (potentiated sulfonamides or enrofloxacin).

Moreover, for those animals that were diagnosed with urolithiasis, 28 underwent a surgical procedure: either cystostomy (*n* = 17), urethrostomy (*n* = 9), or both (*n* = 2). However, in some animals, the urolith passed the urinary tract and was eliminated only with medical treatment. The cases that failed to respond to the standard treatment were given diazepam or midazolam twice a day for 3 to 5 days, since their antispasmodic effect allows the urinary tract muscles to relax and helps to eliminate the uroliths. Moreover, in five cases, retrograde urohydropropulsion was performed, and none of them were submitted to surgery afterward. Four of them responded, with the urolith passing later without complications, while one did not respond and had a later recurrence. Additionally, preventive treatment with glucosamine and potassium citrate was prescribed in 79 (67.5%) and 54 (46.2%) animals, respectively.

Information about the treatment response was available in 114 cases (97.4%): 73 responded well and achieved complete remission, but 41 failed to respond. A potential benefit of the use of potassium citrate was described (*p* = 0.004), and, when surgery was necessary, the most successful type was urethrotomy (*n* = 0.010) (Table 3).

Finally, as preventive measures, owners were advised to provide a diet low in calcium with timothy hay and pellets and a wider variety of fruits and vegetables; to avoid food containing high levels of oxalate, such as spinach, kale, celery, parsley, and strawberries; and to give 10 mL of fluids by mouth daily with a syringe.

## 4. Discussion

Urinary diseases are common in guinea pigs, but the available literature is still scarce and mostly limited to case reports or case studies [10,11,12,13,14]. In addition to the clinical impact on exotic medicine, the study of these disorders and the anatomy and physiology of the urinary tract of this species also has translational research interest, since guinea pigs are considered a suitable urological model [15].

The individual characteristics of our population and its clinical presentation are similar to those of other reported cases [10,16,17,18,19]. It is worth noting that a high frequency of pain was reported on palpation in the cases of urolithiasis, as observed in other studies [10,12,14], as well as in animals later diagnosed with cystitis/UTI. This finding shows the importance of performing a thorough abdominal palpation during the physical examination in order to identify any changes in the urinary tract.

Genetic factors have been suggested as part of the multifactorial etiology of these types of urinary disorders in guinea pigs [20], so we decided to investigate our patients’ family history of urinary disease in order to assess a possible genetic influence. In fact, we found that females who have these urinary disorders are more likely to have a family history than males. Unfortunately, similar results are not reported in the literature, so this is a first description that should encourage more research on this topic.

Among all small mammals, guinea pigs seem to have the greatest tendency to develop urolithiasis [2,6,21] and, in fact, 44.4% of all of our cases were compatible with this diagnosis, which is in line with the prevalence described in another recent study [19]. In addition, an overrepresentation of males was detected, which is in disagreement with some authors who reported a higher frequency in older females instead [20] and even in others who did not identify any sex predisposition [16,22]. To the best of our knowledge, this is the first time that a greater association of male guinea pigs with the disease has been identified. Regarding the location of the uroliths, the most common sites were the bladder and the urethra, as previously described [16,18]. Moreover, the higher frequency of uroliths found in the bladder of males and in the urethra of female guinea pigs is supported by the results reported in another recent retrospective study [18]. The most likely reason is due to the fact that female guinea pigs have a larger urethral diameter than males, allowing calculi to pass from the bladder to urethra, which leads to obstruction in the second region [9]. Unfortunately, we did not have the results to analyze them in terms of their composition since most owners refused this analysis, as happened in other studies [18]. Although this information has minimal or no influence on the treatment protocol, since calcium-based calculi do not respond effectively to dissolution therapy, this analysis must always be carried out in order to clarify the etiopathogenesis of the disease, allowing for the adoption of the necessary preventive measures [2,16]. Our results show that veterinarians should change the way they communicate with the owner regarding the importance of this analysis, focusing on the potential impact that it can have on prevention. Even so, despite the clear limitations, the presence of certain crystals in the urine is an indicator of its potential components, and calcium carbonate crystals were the most commonly identified in our study, as previously reported [16,22,23]. However, contrary to Hawkins et al. (2009), who identified most calculi as being composed solely of calcium carbonate, in our study, a mixed presence of crystals was more frequent, either with calcium oxalate or with struvite.

In relation to cystitis and UTIs, our study agrees with previous findings that suggested that females are more predisposed to these urinary conditions [17]. A probable reason is the fact that there is an anatomical proximity between the anus and the urethral orifice in female guinea pigs, which entails a greater risk of ascending infection and, consequently, bladder inflammation and infection [5]. Another possible reason may have to do with the potential role of female sex hormones in the development of cystitis and UTIs, as already previously suggested [24]. In fact, these may be the predisposing causes responsible for the higher recurrence rate that we recorded in females compared to males. Interestingly, this finding has not yet been described in the current literature. Moreover, a greater recurrence rate was associated with the diagnosis of cystitis/UTI and not with urolithiasis, as expected given what is described in the literature [5,25]. Although there is little information about the prognosis and recurrence rate in animals with cystitis and UTIs, some authors have suggested a higher incidence of a mild and sometimes subclinical form of chronic cystitis with age [17,26,27]. Therefore, chronic cystitis with intermittent acute episodes could justify our results.

Regarding the clinical presentation, most animals showed urinary signs at the time of admission, such as dysuria and stranguria, as typically described [5,25]. These clinical signs were mainly associated with cystitis/UTI, which may also explain their significantly higher frequency in females compared to males. In contrast, most of the non-specific signs, such as anorexia, weight loss, polyuria and polydipsia (PU/PD), gut stasis, and lethargy, were associated with the diagnosis of urolithiasis, which agrees with other authors that reported that this urinary disease can be very subtle and associated with vague clinical signs [5,7]. Most of these non-specific signs were expected, as they are frequently mentioned in the literature, especially for gut stasis, which is a frequent sequel to other diseases that cause pain, discomfort, and anorexia [25]. PU/PD are not common in lower urinary tract diseases, so their manifestation may have had another underlying cause in our population. In fact, two guinea pigs had hydronephrosis, which may have led to the development of chronic kidney disease, and two others had a UTI secondary to diabetes. Therefore, these two metabolic diseases may have been the reason for this clinical manifestation in these animals [7,17]. Interestingly, the presentations of dysuria, hematuria, stranguria, and abdominal pain that are often associated with trauma and inflammation caused by urinary tract calculi were significantly more present in the animals diagnosed with cystitis and/or UTIs, which shows that these findings should not be considered as exclusive indicators of urolithiasis.

Most cases of urolithiasis were diagnosed using radiography, which shows the importance of performing this examination in animals with clinical urinary signs [5,8,18]. Although the ultrasound was used less, its routine use should be encouraged because it allows for the evaluation of the remaining urinary structures and the detection of secondary abnormalities, such as hydronephrosis, hydroureter, and dilated and obstructed regions [10,11,13]. These two complementary tests are the preferred ones to establish this diagnosis, but they also have some limitations. Therefore, when these conventional methods do not allow for a clear visualization of the urinary tract or when it is necessary to assess the degree of ureteral obstruction, the use of an ultrasound-guided percutaneous antegrade pyelography technique has recently been suggested [28]. Moreover, transurethral cystoscopy can be indicated in female guinea pigs for a direct visualization of the urethra and bladder, or even to remove small uroliths (less than 5 mm in diameter) in a faster and less invasive way than the traditional cystotomy [14,29].

Regarding the treatment, more than a quarter of our general population had undergone surgery to treat their urinary disorder, specifically urolithiasis. Urethrotomy showed a significantly higher therapeutic response rate than cystotomy, which remains the gold standard for the surgical treatment of urolithiasis, as suggested in the literature [5,9]. Unfortunately, the number of animals subjected to both procedures was limited and cases of urethrotomy have only been recently described, preventing further conclusions. Nonetheless, this result shows that urethrotomy should be considered in future cases in which the removal of urinary calculi is necessary, enhancing its description and, consequently, the potential confirmation of this interesting finding. In fact, it should be noted that surgical procedures relating to the urinary tract of guinea pigs have advanced significantly in recent years. For example, a penile amputation and pre-scrotal urethrostomy were recently described for the first time in a male guinea pig diagnosed with balanoposthitis/posthitis and urethritis [30]. In addition, as some structural defects (e.g., the presence of urethral diverticulum) can be considered predisposing factors for urolithiasis, surgical procedures such as urethroplasty could also be indicated [12]. In cases where surgery is not possible due to animal or owner constraints, ultrasound-guided percutaneous antegrade hydropropulsion can be performed to relieve ureteral obstruction and the associated clinical signs, as previously suggested [10]. 

There are some preventive treatments described in the literature, although, in our study we only investigated the use of potassium citrate and, for the first time, the influence of glucosamine. According to our results, animals treated with a conventional treatment plus potassium citrate presented a significantly better response to the treatment. This information is interesting and relevant, as it supports the idea that is frequently suggested in the literature regarding the potential effect of this supplementation to bind calcium and decrease its activity [5,8,25]. Therefore, the oral administration of potassium citrate at a dose of 20 mg/kg twice daily has been recommended [8]. On the contrary, the use of glucosamine had no significant influence on the response to treatment. Further studies, particularly of a prospective nature, will be necessary to determine this potential therapeutic impact more accurately, which has not been verified in our population. It would also be interesting to analyze the preventive effect of hydrochlorothiazide as a thiazide diuretic that reduces urinary calcium, potassium, and citrate [8,25]. Its use is reported in the literature with some clinical success, but, as it was only recently recommended for urolithiasis, the number of cases in which it was administered in our hospital was small, preventing it from being analyzed in this study.

Moreover, in terms of preventing the development of these urinary diseases, it is also crucial that guinea pig owners have basic knowledge concerning the hygiene, health, and husbandry care of this species. Fortunately, according to a recent study, it appears that the majority are well-informed about these issues, minimizing risk factors (e.g., poor sanitation, hygiene, and nutrition) and seeking veterinary assistance early when necessary [4]. One way to prevent urolithiasis is to encourage water intake, as it is consequently associated with higher urinary production and less urine retention. According to Balsiger et al. (2017), guinea pigs prefer to drink water from nipple drinkers rather than from open dishes, so the former should be preferred to ensure the intake of a greater amount of water. Unfortunately, the relationship between drinking preferences and the development of urinary disorders could not be addressed in this study due to a lack of available information. In order to assess and confirm this expected association, further studies should assess the drinking habits of guinea pigs and the frequency of urolithiasis. Finally, nutrition also plays an important role, as it suggests an increased risk of urolithiasis in animals fed alfalfa hay, which has higher levels of calcium [20]. Therefore, to prevent crystal precipitation and calculi formation, diets containing timothy or grass hays, pellets, and a variety of vegetables and fruits should be advised, as suggested by the veterinarians who treated our animals [29].

Our study has some limitations that should be mentioned. Firstly, in the majority of the cases, it was not possible to reach a definitive diagnosis between cystitis and a UTI due to the absence of results from the bacteriological culture test that was not performed because of the financial limitations imposed by the owners. Secondly, due to the retrospective nature of the study and the extensive evaluation period, some information could not be collected, mainly from older cases. Lastly, it was not possible to compare our results with a group of healthy animals because, in this species, contrary to dogs and cats, there are no routine appointments (e.g., for vaccination), so when they are assisted in the hospital, it is always because they are sick. 

## 5. Conclusions

This study contributes with new information about the main urinary disorders in guinea pigs, which are poorly described in the literature, despite being common in this species. Our main conclusions are: (1) female guinea pigs are more likely to have a family history of urinary disease, to present dysuria and stranguria at admission, and to have recurrence compared to males; (2) male guinea pigs are more predisposed to urolithiasis, and females to cystitis/UTI; (3) guinea pigs diagnosed with cystitis and/or a UTI are more likely to have clinical urinary signs (dysuria, hematuria, and stranguria) and abdominal pain on palpation compared to those diagnosed with urolithiasis; (4) the use of potassium citrate, in addition to conventional treatments, and the performance of urethrotomy instead of other surgical approaches are associated with a better therapeutic response.

In addition, our study shows that it is still necessary to make owners and veterinarians aware of the clinical importance of a complete diagnostic approach whenever a lower urinary tract disease is suspected in guinea pigs.

## Figures and Tables

**Table 1:** Association between patient and diagnostic features with the guinea pigs’ sex (female versus male) in 117 clinical cases diagnosed with lower urinary tract disease.

	Sex				
	Female		Male		*p*-Value
	*n*	%	*n*	%	
**Reproductive condition (*n* = 117)**					*p* < 0.001 (S)
Intact	67	65.00%	36	35.00%	
Neutered	1	7.10%	13	92.90%	
**Type of medical consultation (*n* = 117)**					*p* = 0.091 (NS)
First opinion	43	65.20%	23	34.80%	
Referral	25	49.00%	26	51.00%	
**Disease recurrence (*n* = 117)**					*p* = 0.038 (S)
Yes	42	67.70%	20	32.30%	
No	26	47.30%	29	52.70%	
**Family history of UD (*n* = 117)**					*p* < 0.001 (S)
Yes	45	75.00%	15	25.00%	
No	23	40.40%	34	59.60%	
**Reason for consultation (*n* = 117)**					*p* = 0.337 (NS)
Urinary signs	49	59.80%	33	40.20%	
Urinary + Nonspecific signs	12	66.70%	6	33.30%	
Nonspecific signs	7	43.80%	9	56.20%	
Shock	0	0.00%	1	100.00%	
**Dysuria (*n* = 117)**					*p* = 0.038 (S)
Yes	54	64.30%	30	35.70%	
No	14	42.40%	19	57.60%	
**Hematuria (*n* = 117)**					*p* = 0.075 (NS)
Yes	50	64.10%	28	35.90%	
No	18	46.20%	21	53.80%	
**Stranguria (*n* = 117)**					*p* = 0.038 (S)
Yes	54	64.30%	30	35.70%	
No	14	42.40%	19	57.60%	
**Abdominal pain (*n* = 117)**					*p* = 0.126 (NS)
Yes	46	63.90%	26	36.10%	
No	22	48.90%	23	51.10%	
**Diagnosis (*n* = 117)**					*p* < 0.001 (S)
Urolithiasis	20	38.50%	32	61.50%	
Cystitis/UTI	48	73.80%	17	26.20%	

*n*—number of guinea pigs; *p*—statistical significance; S—statistically significant association; NS—non-statistically significant association; UD—urinary disease; US—Urinary signs; UTI—urinary tract infection; %—relative percentage of guinea pigs.

**Table 2:** Association between patient and diagnostic features with the guinea pigs’ diagnosis (urolithiasis, cystitis, and/or UTI) in 117 clinical cases diagnosed with lower urinary tract disease.

	Urolithiasis		Cystitis/UTI		*p*-Value
	*n*	%	*n*	%	
**Sex (*n* = 117)**					*p* < 0.001 (S)
Female	20	29.40%	48	70.60%	
Male	32	65.30%	17	34.70%	
**Reproductive condition (*n* = 117)**					*p* = 0.393 (NS)
Intact	44	42.70%	59	57.30%	
Neutered	8	57.10%	6	42.90%	
**Type of medical consultation (*n* = 117)**					*p* = 0.454 (NS)
First opinion	27	40.90%	39	59.10%	
Referral	25	49.00%	26	51.00%	
**Disease recurrence (*n* = 117)**					*p* = 0.006 (NS)
Yes	20	32.30%	42	67.70%	
No	32	58.20%	23	41.80%	
**Family history of UD (*n* = 117)**					*p* = 0.196 (NS)
Yes	23	38.30%	37	61.70%	
No	29	50.90%	28	49.10%	
**Reason for consultation (*n* = 117)**					*p* = 0.002 (S)
Urinary signs	27	32.90%	55	67.10%	
Urinary + Nonspecific signs	13	72.20%	5	27.80%	
Nonspecific signs	111	68.80%	5	31.30%	
Shock		100.00%	0	0.00%	
**Dysuria (*n* = 117)**					*p* = 0.027 (S)
Yes	32	38.10%	52	61.90%	
No	20	60.60%	13	39.40%	
**Hematuria (*n* = 117)**					*p* < 0.001 (S)
Yes	24	30.80%	54	69.20%	
No	28	71.80%	11	28.20%	
**Stranguria (*n* = 117)**					*p* = 0.038 (S)
Yes	32	38.10%	52	61.90%	
No	20	60.60%	13	39.40%	
**Abdominal pain (*n* = 117)**					*p* = 0.004 (S)
Yes	24	33.30%	48	66.70%	
No	28	62.20%	17	37.80%	

*n*—number of guinea pigs; *p*—statistical significance; S—statistically significant association; NS—non-statistically significant association; UD—urinary disease; US—Urinary signs; UTI—urinary tract infection; %—relative percentage of guinea pigs.

**Table 3:** Association between diagnosis and therapeutic features with the guinea pigs’ response to treatment in 114 clinical cases diagnosed with lower urinary tract disease.

	Response to Treatment				
	Yes		No		*p*-Value
	*n*	%	*n*	%	
**Diagnosis (*n* = 114)**					*p* = 0.994 (NS)
Urolithiasis	32	64.00%	18	36.00%	
Cystitis/UTI	41	64.10%	23	35.90%	
**Type of treatment (*n* = 114)**					*p* = 0.520 (NS)
Medical	50	61.70%	31	38.30%	
Surgical	23	69.70%	10	30.30%	
**Use of glucosamine (*n* = 114)**					*p* = 0.408 (NS)
Yes	52	66.70%	26	33.30%	
No	21	58.30%	15	41.70%	
**Use of potassium citrate (*n* = 114)**					*p* = 0.004 (S)
Yes	40	78.40%	11	21.60%	
No	33	52.40%	30	47.60%	
**Type of surgery (*n* = 28)**					*p* = 0.010 (S)
Cystotomy	10	58.80%	7	41.20%	
Urethrotomy	9	100.00%	0	0.00%	
Cystotomy + Urethrotomy	0	0.00%	2	100.00%	

*n*—number of guinea pigs; *p*—statistical significance; S—statistically significant association; NS—non-statistically significant association; UD—urinary disease; US—Urinary signs; UTI—urinary tract infection; %—relative percentage of guinea pigs.

## Data Availability

The data that support the findings of this retrospective study are available upon reasonable request to the authors.
